# Comparative analysis of microRNA profiles between adult *Ascaris lumbricoides* and *Ascaris suum*

**DOI:** 10.1186/1746-6148-10-99

**Published:** 2014-04-27

**Authors:** Chang-Chun Shao, Min-Jun Xu, Samer Alasaad, Hui-Qun Song, Lifei Peng, Jian-Ping Tao, Xing-Quan Zhu

**Affiliations:** 1 College of Veterinary Medicine, Yangzhou University, Yangzhou, Jiangsu Province 225009, PR China; 2 State Key Laboratory of Veterinary Etiological Biology, Key Laboratory of Veterinary Parasitology of Gansu Province, Lanzhou Veterinary Research Institute, Chinese Academy of Agricultural Sciences, Lanzhou, Gansu Province 730046, PR China; 3 Estación Biológica de Doñana, Consejo Superior de Investigaciones Científicas (CSIC), Avda. Américo Vespucio s/n 41092, Sevilla, Spain; 4 Department of Parasitology & Clinical Parasitology, Guangdong Medical College, Zhanjiang, Guangdong Province 524023, PR China

**Keywords:** MicroRNA (miRNA), *Ascaris lumbricoides*, *Ascaris suum*, Comparative analysis

## Abstract

**Background:**

The parasitic nematodes *Ascaris lumbricoides* and *A. suum* are of great public health and economic significance, and the two taxa were proposed to represent a single species. miRNAs are known with functions of gene regulations at post-transcriptional level.

**Results:**

We herein compared the miRNA profiles of *A. lumbricoides* and *A. suum* female adults by Solexa deep sequencing combined with bioinformatics analysis and stem-loop real-time PCR. Using the *A. suum* genome as the reference genome, we obtained 171 and 494 miRNA candidates from *A. lumbricoides* and *A. suum*, respectively. Among which, 74 miRNAs were shared between the two taxa, 97 and 420 miRNAs were *A. lumbricoides* and *A. suum* specific. Target and function prediction revealed a significant set of targets which are related to ovarian message protein, vitellogenin and chondroitin proteoglycan of the two nematodes. Enrichment analysis revealed that the percentages of most predicted functions of the miRNA targets were similar, with some taxon specific or taxon enhanced functions, such as different target numbers, specific functions (NADH dehydrogenase and electron carrier functions), etc.

**Conclusions:**

This study characterized comparatively the miRNAs of adult *A. lumbricoides* and *A. suum,* and the findings provide additional evidence that *A. lumbricoides* and *A. suum* represent a single species. Due to the fast evolution nature of miRNAs and the different parasitic living conditions of humans and pigs, the phenomenon above might indicate a fast evolution of miRNAs of *Ascaris* in humans and pigs.

## Background

Roundworms *Ascaris lumbricoides* and *A. suum* are important parasites of human and pig health and socio-economic significance, with global distribution [1,2]. *Ascaris* infects approximately 1.2 billion people globally and has been associated with intestinal pathology, respiratory symptoms and malnutrition in children from endemic areas [3]. It was reported that humans and pigs can both be infected with the two nematodes [2]. There are very limited nucleotide differences in the sequences of internal transcribed spacers (ITS) of ribosomal DNA between the two ascarid species [4]. *A. lumbricoides* and *A. suum* are now proposed to represent a single species, and *A. suum* is considered a synonym of *A. lumbricoides*[5]. The genome and transcripts of *A. suum* were recently available, which provided valuable resources for better understanding and further studies of the biology of the *Ascaris* parasites [6-8].

MicroRNAs (miRNAs) are non-coding small RNA of 18–24 nt in length. They are considered as key regulators for gene expression at the post-transcriptional level [9-12]. Due to their key regulating functions in growth, metabolism, development and cell differentiation and their ability to respond to environmental and developmental signals, miRNA is essential for the complex life cycles of human and animal parasites [13-22]. miRNAs are also important for pathogen-host interactions [23-26]. Recently studies have indicated that miRNAs may represent potential biomarkers for various biomedical problems, such as the differentiation of different tumors and muscular tissues [27-29], and they may provide biomarkers for the characterization of different genotypes of *Toxoplasma gondii*[30].

There might be differences in gene expression and regulation given the different living environments of *A. lumbricoides* and *A. suum,* although they are considered to represent the same species. Therefore, it would be interesting to characterize the expression profiles of miRNAs in the two taxa. Herein the objective of the present study was to examine and compare the miRNA profiles of *A. lumbricoides* and *A. suum* using an integrative approach combining Solexa deep sequencing combined with bioinformatics analysis and stem-loop real-time PCR.

## Methods

### Ethics statement

The present study was approved by the Ethics Committee of Lanzhou Veterinary Research Institute, Chinese Academy of Agricultural Sciences (Approval No. LVRIAEC2011-006), and the *A. lumbricoides* and *A. suum* samples were collected strictly according to the requirements of the Ethics Procedures and Guidelines of the People's Republic of China.

### Nematodes

Female adults of *A. suum* roundworms were obtained from slaughtered pigs in Shenzhen, China. Female adults of *A. lumbricoides* were obtained from a patient with ascariasis after being treated with piperazine in Zhanjiang, Guangdong Province, China. Worms were incubated in physiological saline for 3 h at 37°C and then washed 3 times to get rid of contamination from hosts. Female adults were identified morphologically and their identity was further ascertained by PCR amplification and sequencing of the first internal transcribed spacer (ITS-1) of rDNA [4]. The nematodes were stored at −80°C until use.

### Total RNA isolation, small RNA preparation and deep sequencing

Total RNA was prepared from a whole single adult of *A. lumbricoides* and *A. suum* respectively, using Trizol reagent (Invitrogen) according to the manufacturer’s protocol. Ten μg total RNA and Novex 15% TBE-Urea gel (Invitrogen) were used for small RNA isolation. The RNA fragments of 18–30 bases long were added with 5’ and 3’ adaptors (Illumina) to the both ends, reverse transcripted with a RT-PCR kit (Invitrogen), and sequenced employing a Solexa sequencer at Huada Genomics Institute Co. Ltd, China.

### Computational analysis

Adaptors, low quality sequences and reads smaller than 18 nt were removed from the raw sequencing data. The reads were then searched against GenBank and Rfam database ( http://www.sanger.ac.uk/software/Rfam) to identify non-coding RNA. The remaining reads were mapped onto the *A. suum* genome by using the SOAP [31] with the sequences of pre-miRNA meeting the three criteria: 1) there was a standard stem-loop structure of pre-miRNA; 2) mature miRNAs were present in one arm instead of the loop of hairpin precursors; and 3) the free energy hybridization was lower than −18 kcal/mol. The identified miRNA candidates were searched against the known miRNAs of *A. suum* deposited in the Sanger miRBase with Blast [32]. Unmatched miRNA candidates were marked as novel miRNA. Targets of miRNA candidates were predicated with RNAhybrid [33] with the following extra parameters: A) the △△G was set as lower than −25 kcal/mol; B) *P*-value was set as ≤ 0.01. The Gene Ontology database (GO, http://www.geneontology.org/), Blast and Interproscan ( http://www.ebi.ac.uk/Tools/pfa/iprscan/) were used for prediction of functions of predicted targets.

### Analysis of novel miRNA expression

The representative novel miRNA in the two nematodes were certified using stem-loop real-time reverse transcription polymerase chain reaction (RT-PCR) with SYBR Green [34]. The house keeping gene β-actin of *A. suum* (GenBank accession no. BI594141) was used as the endogenous control with primers as follows: forward primer (5′-CTCGAAACAAGAATACGATG-3′) and reverse primer (5′-ACATGTGCCGTTGTATGATG-3′). Primers were synthesized by Shenggong Co, Ltd., China. The cycle conditions were as follows: 94°C for 30s, 52°C for 30s, 72°C for 30s and finally with a single extension at 72°C for 10 min. The quantification of each miRNA relative to β-actin gene was calculated using the equation: N = 2^-ΔCt^, ΔCt = Ct_miRNA_-Ct_acin_[35].

## Results

### Profile differences in short RNAs between the two *Ascaris* taxa

Deep sequencing yielded 18.29 and 11.72 million raw reads in adult of *A. lumbricoides* and *A. suum*, respectively, with 14.69 and 9.76 million high quality reads that longer than 18 nt. Length distribution analysis showed that the reads of *A. lumbricoides* and *A. suum* were focused on 21–23 nt. Among the clean reads, 46.11% and 39.97% were identified as non-coding RNA (ncRNA) in *A. lumbricoides* and *A. suum* respectively, including tRNA, rRNA, snRNA and snoRNA, which were at near the same level. Among the high quality reads, 72.47% of the total reads were shared by the two parasites (Table 1), while 1.71 and 1.08 million was *A. lumbricoides* and *A. suum* specific.

**Table 1:** **Common and taxon-specific reads of *Ascaris lumbricoides *** **and *Ascaris suum***

**Classification**	**Unique sRNAs (%)**	**Total sRNAs (%)**
Total sRNAs	2917391 (100%)	24449036 (100%)
Common reads^a^	126807 (4.35%)	17718517 (72.47%)
*A. lumbricoides* specific^b^	1711536 (58.67%)	4379273 (17.91%)
*A. suum* specific^c^	1079048 (36.99%)	2351246 (9.62%)

***Note***: ^a^reads that were shared by the two taxa; ^b^reads found in *A. lumbricoides*, but not found in *A. suum*; ^c^reads found in *A. suum*, but not found in *A. lumbricoides*.

### miRNA profiles of the two taxa

By mapping onto the *A. suum* genome, we obtained 171 and 494 miRNA candidates, with precursors meeting the criteria listed in the method and having standard stem-loop structures (Table 2, Additional file 1: Table S1). Among the miRNA candidates, 68 and 87 miRNAs were matched with the known *A. suum* miRNAs deposited in the miRBase database, and 63 of them were shared. The left miRNA candidates were marked as novel miRNAs, and among which only 11 miRNAs were shared, therefore, 92 and 396 miRNAs were *A. lumbricoides* and *A. suum* specific. Totally, there were 74 miRNAs shared by the two nematodes, including 63 known miRNA and 11 novel ones; and there were 97 and 420 miRNAs being *A. lumbricoides* and *A. suum* specific.

**Table 2:** **Comparison of miRNA profiles in *Ascaris lumbricoides *** **(Alu) and *A. suum *** **(Asu)**

	**Shared**	**Asu-specific**	**Alu-specific**	**Asu-total**	**Alu-total**
Novel	11	396	92	407	103
Known	63	24	5	87	68
Total	74	420	97	494	171

### Target prediction and the functional prediction of the predicted targets

A total of 57,359 mRNA and EST items of *Ascaris* deposited in NCBI were downloaded and used for target prediction. Under the stringent matching criteria, it was found that the target numbers of both nematodes ranged from one to thousands. For *A. lumbricoides*, the target number ranged from one (Alu-miR-novel-012-3p, Alu-miR-novel-066-5p, Alu-miR-novel-102-3p) to 3,562 (Alu-miR-novel-063-3p), 255 in average. For *A. suum*, the target number ranged between one (asu-miR-novel-027-3p, asu-miR-novel-044-5p, and asu-miR-novel-068-5p) and 3,343 (asu-miR-novel-039-3p), 168 in average.

Functional prediction revealed a significant set of targets which are related to ovarian message protein in *A. lumbricoides* (n = 9) and *A. suum* (n = 10)*,* a set of vitellogenin (5 in *A. lumbricoides* and 18 in *A. suum*), and a set of chondroitin proteoglycan (5 in *A. lumbricoides* and 7 in *A. suum*). The same phenomenon is found in NADH dehydrogenase (n = 7 in *A. suum*, n = 3 in *A. lumbricoides*), 40s ribosomal protein (n = 14 in *A. suum*, n = 2 in *A. lumbricoides*), and 60s ribosomal protein (n = 11 in *A. suum*, n = 7 in *A. lumbricoides*). As a distinguished character, there were more targets related to movement in *A. suum*, such as actin (n = 5) and tubulin (n = 6), while such targets were very few in *A. lumbricoides*.

**Figure 1:**
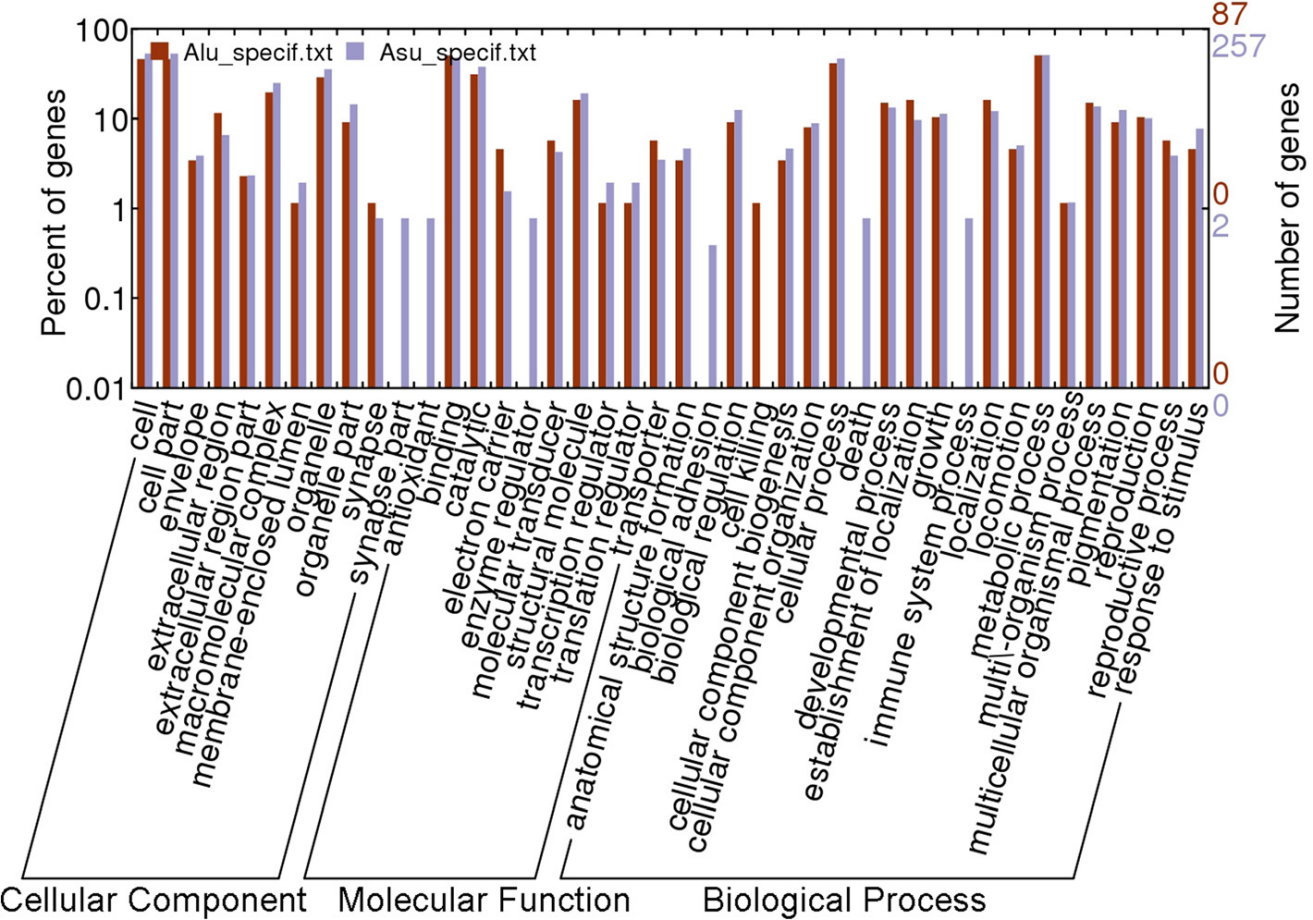
**Enrichment analysis of the functions of strain-specific miRNAs targets of *Ascaris suum *and *A*. *lumbricoides.*** The horizontal axis: Gene Ontology analysis to the targets, including cellular component, molecular function, and biological process. The vertical axis: percentage of genes in total targets.

Gene Ontology (GO) analysis of the targets of miRNAs resulted in 3 respect outcomes, including cellular component, molecular function and biological process. Enrichment analysis showed that percentages of most targets functions of *A. suum* and *A. lumbricoides* were similar, except a few items (Figure 1), which indicated a very closely metabolism pattern of the two parasites. However, for cell component, miRNA targets of *A. suum* had an extra cellular component part named as “synapse part”; for molecular function, one function named “electron carrier” was only found in targets of *A. suum*. In addition, three biological processes named biological adhesion, death, and immune system process were only found in the miRNA targets of *A. suum*.

### Quantification of *A. lumbricoides* and *A. suum* miRNAs

**Figure 2:**
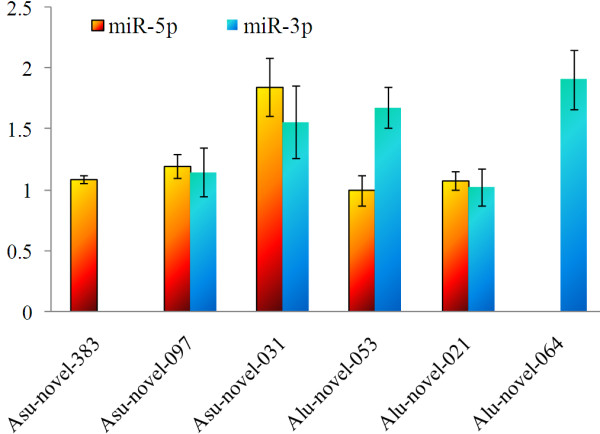
**The main blast result and standard stem-loop structure of precursors of one of the representative miRNAs.** Upper: plain text sequence of precursor of asu-miR-share-031 and the main blast result with mature miRNA matched; Down: stem-loop structure of the precursor. Detailed blast information was shown in (Additional file 2: Figure S1).

**Figure 3:**
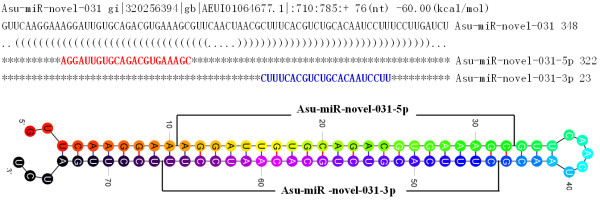
**Quantification of representative miRNAs of *Ascaris lumbricoides *and *A*. *suum*.** Three novel miRNAs of each nematode were detected. Two miRNAs named asu-miR-novel-031 and alu-miR-novel-53 had significant expression differences of 5p and 3p mature miRNAs. The alu-miR-novel-64-5p and asu-miR-novel-383-3p were not successfully amplified, which might due to their low expression levels.

Three *A. suum* and *A. lumbricoides* specific novel miRNAs, including asu-miR-novel-383, asu-miR-novel-097 and asu-miR-novel-031 in *A. suum,* and alu-miR-novel-053, alu-miR-novel-021, and alu-miR-novel-064 in *A. lumbricoides*, were representatively selected for quantification using modified stem-loop qRT-PCR (Figure 2). These 6 miRNAs had the lowest △G energy, and/or more matched variants, and had mature miRNAs at both of the arms of the stem-loop precursors. The representative blast and standard stem-loop structure as asu-miR-novel-031 was shown in Figure 3. Detailed blast information with variants of others was shown in Additional file 2.

Both of the mature miRNAs of the 6 selected miRNAs can be successfully detected, except the alu-miR-novel-64-5p and asu-miR-novel-383-3p. Two miRNAs named asu-miR-novel-031 and alu-miR-novel-53 had significant expression differences at 5p and 3p. For alu-miR-novel-53, the expression levels were 1.00 ± 0.12 at 5p, and it was 1.68 ± 0.17 at 3p. And for asu-miR-novel-031, the relative expression level was 1.85 ± 0.24 at 5p, while it was 1.56 ± 0.30 at 3p. The alu-miR-novel-64-5p and asu-miR-novel-383-3p were not successfully amplified, which could be attributed to the very low expression levels, despite that the primers and reaction mixture were modified several times.

## Discussion

A previous study revealed that *A. lumbricoides* and *A. suum* have identical 5.8S and ITS-2 rDNA sequences [4]. There were only six (1.3%) nucleotide differences in ITS-1. All these genetic evidences supported the proposal that *A. lumbricoides* and *A. suum* represent a single species [4,5].

Due to their specific expression in different organisms, tissues and cells, miRNAs may provide potential novel biomarkers [27-29]. Our previous study indicated that different *T. gondii* genotypes have their unique miRNA profiles [30]. In the present study, the two nematode taxa shared very high percentage of total reads (72.47%), while having very small percentage of unique reads (4.35%), and the total percentage of ncRNA was at similar level (46.11% and 39.97% in *A. lumbricoides* and *A. suum*, respectively), which indicated huge redundance of some ncRNA of the two nematodes. When parasitic environments change, the gene expression of parasites will be correspondingly changed, and regulators such as miRNAs will also be changed, therefore the death and new creation of miRNAs are very common [36]. For the different parasitic environment of pigs and humans, adaptive modifications should have occurred. So although the sRNA/shared-miRNAs for fundamental metabolism were similar in the two taxa, there still are some specific miRNAs in each nematode taxon. We obtained 171 and 494 miRNA candidates as expression profiles, with 74 miRNAs being shared by the two nematode taxa, and 97 and 420 miRNAs being *A. lumbricoides* and *A. suum* specific. The same precursors of a miRNA can be found in the genome of *A. lumbricoides* or *A. suum*, however, we obtained fewer miRNAs from *A. lumbricoides* than from *A. suum*, which indicated that some miRNAs in *A. lumbricoides* were not expressed. This difference might be resulted from the different parasitic living environment in pigs and humans.

Function prediction and enrichment analysis showed that targets of the miRNAs of the two parasites had similar metabolism patterns, including cellular component, molecular function and biological process, with some specific differences, such as different target number, specific functions (NADH dehydrogenase and electron carrier functions), etc.

## Conclusion

The present study characterized comparatively the miRNAs of adult *A. lumbricoides* and *A. suum,* and the findings support the recent proposal that *A. lumbricoides* and *A. suum* represent a single species [5]. Due to the fast evolution nature of miRNAs and the different parasitic living conditions of humans and pigs, the phenomenon above might indicate a fast evolution of miRNAs of *Ascaris*.

## Competing interests

The authors declare that they have no competing interests.

## Authors’ contributions

XQZ, MJX and JPT conceived and designed the experiment, and critically revised the manuscript. CCS, MJX, SA and HQS performed the experiments, analyzed the data, and drafted the manuscript. LP helped in the study design, study implementation, and manuscript revision. All authors read and approved the final manuscript.

## Supplementary Material

Additional file 1: Table S1 Shared and strain-specific miRNA with their predicated target of *Ascaris lumbricoides* and *A. suum*. Click here for file

Additional file 2: Figure S1 Detailed blast results of variants and precursors of representative miRNAs in *Ascaris lumbricoides* and *A. suum*. Click here for file
