# Supplementary material for: Comparative analysis of microRNA profiles between adult Ascaris lumbricoides and Ascaris suum
Source: BMC Vet Res. 2014 Apr 27;10:99. doi: 10.1186/1746-6148-10-99 (PMC4021693; doi:10.1186/1746-6148-10-99)

Additional file 2. Detailed blast result of variants and precursors of representative miRNAs

1) Alu-miR-novel-064

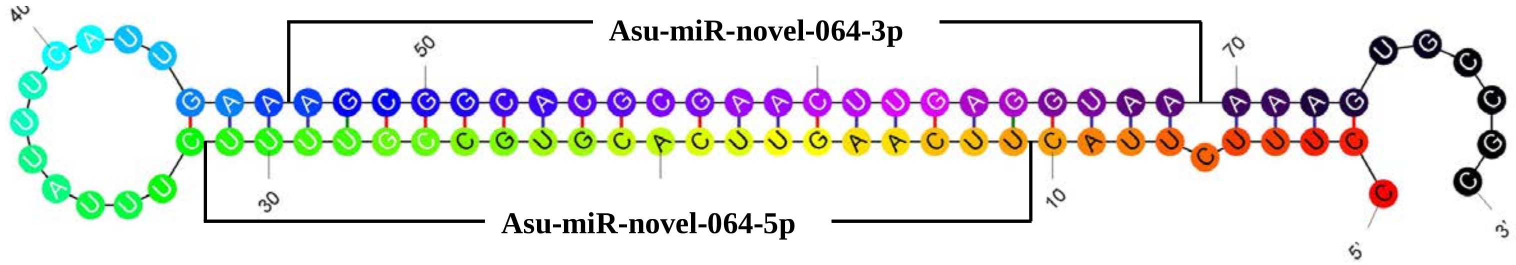

gi|320306869|gb|AEUI01014202.1|:2242:2320:- 79(nt) -40.90(kcal/mol)

CCUUUCUUACUUCAAGUUCACGUGCCGUUUUCUUUAUUUCAUUGAAAGCGGCACGCGAACUUGAGGUAAAAAGUGCCGC Alu-miR-novel-064 253

.((((.(((((((((((((.((((((((((((...........))))))))))))).)))))))))))))))))......

**********UUCAAGUUCACGUGCCGUUUUC*** Alu-miR-novel-064-5p 1

AGCGGCACGCGAACUUGAGGUAA******** Alu-miR-novel-064-3p 252

----------UUCAAGUUCACGUGCCGUUUUC--- t1398356 1

---AAGCGGCACGCGAACUUGAGG------------- t1006145 1

---AAGCGGCACGCGAACUUGAGGU------------ t1053888 1

---AAGCGGCACGCGAACUUGAGGUA----------- t0138760 4

---AAGCGGCACGCGAACUUGAGGUAA---------- t0068853 10

---AAGCGGCACGCGAACUUGAGGUAAA--------- t0291065 2

--AGCGGCACGCGAACUUGAG-------------- t0109393 5

--AGCGGCACGCGAACUUGAGG------------- t0240431 2

--AGCGGCACGCGAACUUGAGGU------------ t0155669 3

--AGCGGCACGCGAACUUGAGGUA----------- t0029787 35

--AGCGGCACGCGAACUUGAGGUAA---------- t0010146 165

```
----------------------------------------------AGCGGCACGCGAACUUGAGGUAAA--------- t0102921 6
-----------------------------------------------GCGGCACGCGAACUUGAGGUA----------- t0252968 2
-----------------------------------------------GCGGCACGCGAACUUGAGGUAA---------- t0057453 13
-----------------------------------------------GCGGCACGCGAACUUGAGGUAAA--------- t0630265 1
------------------------------------------------CGGCACGCGAACUUGAGGUAA---------- t0263691 2
```

2) Alu-miR-novel-053

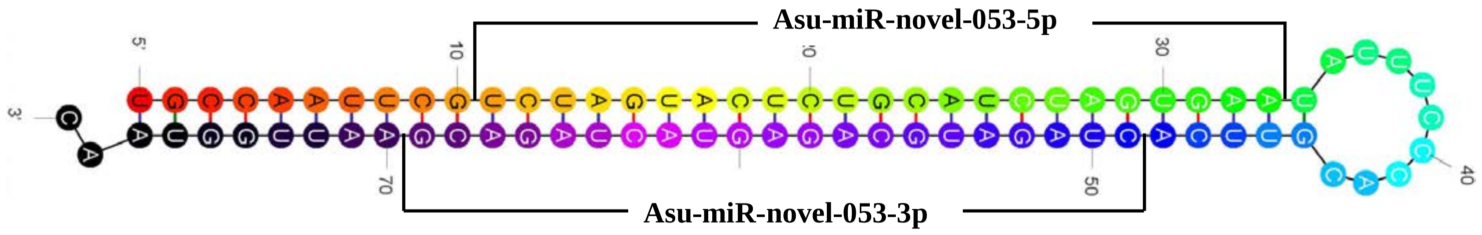


```
gi|320297968|gb|AEUI01023103.1|:1933:2011:- 79(nt) -58.80(kcal/mol)
UGCCAAUUCGUCUAGUACUCUGCAUCUAGUGAAUAUUUCCCACGUUCACUAGAUGCAGAGUACUAGACGAAUUGGUAAC Alu-miR-novel-053 23
((((((((((((((((((((((((((((((((((.........)))))))))))))))))))))))))))))))))).. 
**********UCUAGUACUCUGCAUCUAGUGAA********************************************** Alu-miR-novel-053-5p 7
************************************************CUAGAUGCAGAGUACUAGACG********** Alu-miR-novel-053-3p 16
----------UCUAGUACUCUGCAUCUAGUGA----------------------------------------------- t0476183 1
----------UCUAGUACUCUGCAUCUAGUGAA---------------------------------------------- t0120476 5
----------UCUAGUACUCUGCAUCUAGUGAAU--------------------------------------------- t0515075 1
-----------------------------------------------ACUAGAUGCAGAGUACUA-------------- t1272648 1
-----------------------------------------------ACUAGAUGCAGAGUACUAGACG---------- t1631272 1
------------------------------------------------CUAGAUGCAGAGUACUAGA------------ t1370274 1
------------------------------------------------CUAGAUGCAGAGUACUAGAC----------- t1157625 1
------------------------------------------------CUAGAUGCAGAGUACUAGACG---------- t0062033 12
```

3) Alu-miR-novel-021

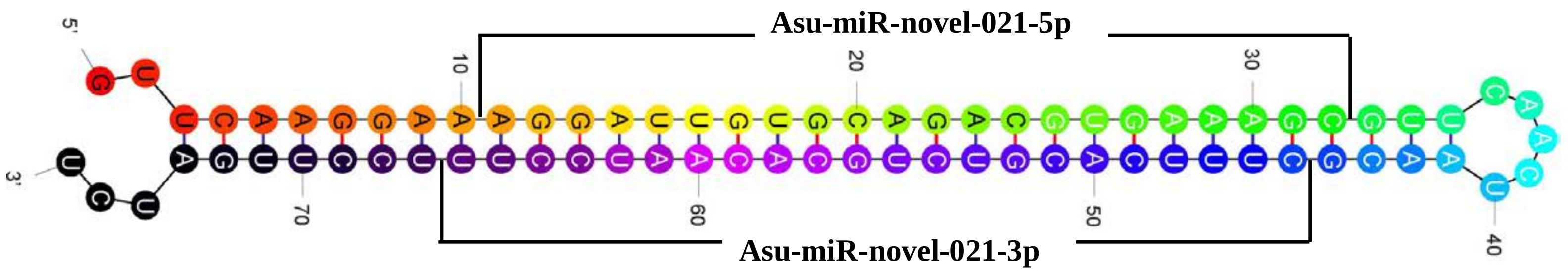


```
gi|320256394|gb|AEUI01064677.1|:710:785:+ 76(nt) -60.00(kcal/mol)
GUUCAAGGAAAGGAUUGUGCAGACGUGAAAGCGUUCAACUAACGCUUUCACGUCUGCACAAUCCUUUCCUUGAUCU Alu-miR-novel-021 33
..(((((((((((((((((((((((((((((((((.....)))))))))))))))))))))))))))))))))...
**********AGGAUUGUGCAGACGUGAAAGC******************************************** Alu-miR-novel-021-5p 18
********************************************CUUUCACGUCUGCACAAUCCUU********** Alu-miR-novel-021-3p 15
--------AAAGGAUUGUGCAGACGUGAAAG--------------------------------------------- t0238133 2
----------AGGAUUGUGCAGACGUGAAAG--------------------------------------------- t0309912 2
----------AGGAUUGUGCAGACGUGAAAGC-------------------------------------------- t0080225 8
----------AGGAUUGUGCAGACGUGAAAGCG------------------------------------------- t0113453 5
-----------GGAUUGUGCAGACGUGAAAGC-------------------------------------------- t0730850 1
--------------------------------------------CUUUCACGUCUGCACAAUC------------- t0137646 4
--------------------------------------------CUUUCACGUCUGCACAAUCC------------ t0183913 3
--------------------------------------------CUUUCACGUCUGCACAAUCCU----------- t0349434 2
--------------------------------------------CUUUCACGUCUGCACAAUCCUU---------- t0131463 4
--------------------------------------------CUUUCACGUCUGCACAAUCCUUU--------- t0361197 1
---------------------------------------------UUUCACGUCUGCACAAUCCUUU--------- t0759185 1
```

4) Asu-miR-novel-031

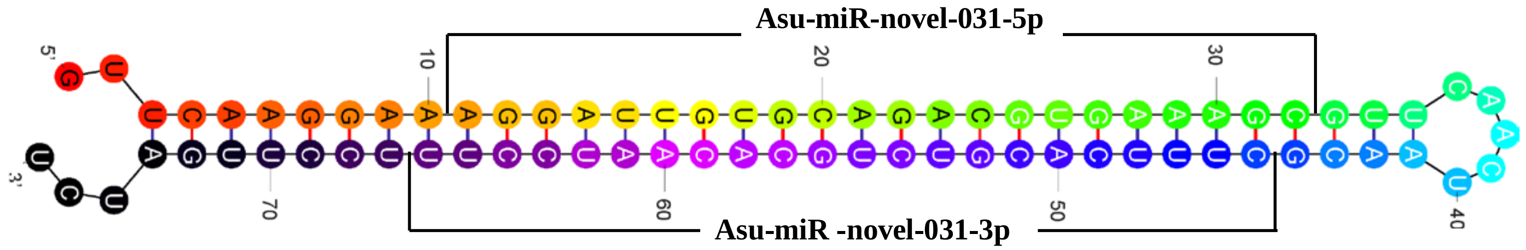

gi|320256394|gb|AEUI01064677.1|:710:785:+ 76(nt) -60.00(kcal/mol)

GUUCAAGGAAAGGAUUGUGCAGACGUGAAAGCGUUCAACUAACGCUUUCACGUCUGCACAAUCCUUUCCUUGAUCU Asu-miR-novel-031 348

..(((((((((((((((((((((((((((((((((.....)))))))))))))))))))))))))))))))))...

**********AGGAUUGUGCAGACGUGAAAGC** Asu-miR-novel-031-5p 322

CUUUCACGUCUGCACAAUCCUU******** Asu-miR-novel-031-3p 23

----AAGGAAAGGAUUGUGCAGACG--- t0516331 1

----AAGGAAAGGAUUGUGCAGACGU-- t0392159 2

-------GAAAGGAUUGUGCAGACGUGAAA-- t0450014 1

-------GAAAGGAUUGUGCAGACGUGAAAG--- t0647302 1

---------AAGGAUUGUGCAGACGUGAAAG--- t1126127 3

---------AAGGAUUGUGCAGACGUGAAAGC-- t0283893 2

----------AGGAUUGUGCAGACGUGAAAG--- t0066673 7

----------AGGAUUGUGCAGACGUGAAAGC-- t0002997 251

----------AGGAUUGUGCAGACGUGAAAGCG--- t0011867 55

-----------GGAUUGUGCAGACGUGAAAGC-- t0249690 2

--CUUUCACGUCUGCACAAUCCU----------- t0395339 2

--CUUUCACGUCUGCACAAUCCUU---------- t0040505 12

--CUUUCACGUCUGCACAAUCCUUU--------- t0061130 7

---UUUCACGUCUGCACAAUCCUUU--------- t0253714 2

5) Asu-miR-novel-097

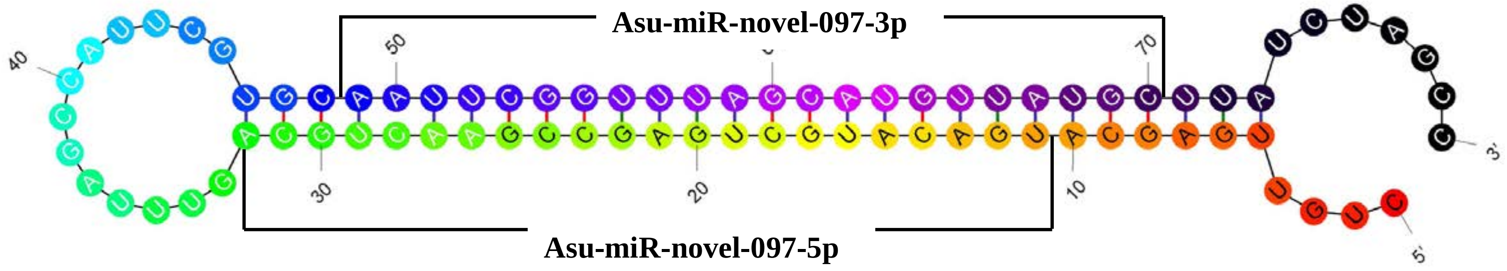


```
gi|320281819|gb|AEUI01039252.1|:345:424:+ 80(nt) -40.32(kcal/mol)
CUGUUGAGCAUGACAUGCUGAGCCGAACUGCAGUUUAGCCAUUCGUGCAAUUCGGUUUAGCAUGUUAUGCUUAUCUAGCC Asu-miR-novel-097 69
....(((((((((((((((((((((((.((((.............)))).)))))))))))))))))))))))).......
**********UGACAUGCUGAGCCGAACUGCA************************************************ Asu-miR-novel-097-5p 43
************************************************AAUUCGGUUUAGCAUGUUAUGC********** Asu-miR-novel-097-3p 26
--------CAUGACAUGCUGAGCCGAACUGC------------------------------------------------- t0599229 1
---------AUGACAUGCUGAGCCGAACUG-------------------------------------------------- t0321728 2
---------AUGACAUGCUGAGCCGAACUGC------------------------------------------------- t0089568 5
---------AUGACAUGCUGAGCCGAACUGCA------------------------------------------------ t0042026 12
----------UGACAUGCUGAGCCGAACUGC------------------------------------------------- t0342107 2
----------UGACAUGCUGAGCCGAACUGCA------------------------------------------------ t0026581 20
----------UGACAUGCUGAGCCGAACUGCAG----------------------------------------------- t1168842 1
-----------------------------------------------CAAUUCGGUUUAGCAUGUUAUGC---------- t0053541 9
------------------------------------------------AAUUCGGUUUAGCAUGUUAUGC---------- t0048730 10
-------------------------------------------------AUUCGGUUUAGCAUGUUAUGC---------- t0113289 4
-------------------------------------------------AUUCGGUUUAGCAUGUUAUGCU--------- t0172298 3
```

6) Asu-miR-novel-383

gi|320319009|gb|AEUI01002062.1|:5885:5957:+ 73(nt) -60.80(kcal/mol)

UUGUGGGAGCGGAGUUAAGUGUCGUUUGUGUCCGAGGCGGACACAAACGACACUUAACACCGCUCCCACAACG Asu-miR-novel-383 10

(((((((((((.((((((((((((((((((((((...)))))))))))))))))))))).))))))))))))..

**********GGAGUUAAGUGUCGUUUGUGUCC*** Asu-miR-novel-383-5p 9

ACAAACGACACUUAACACCGC******** Asu-miR-novel-383-3p 1

----------GGAGUUAAGUGUCGUUUGUGU-- t0167677 3

----------GGAGUUAAGUGUCGUUUGUGUCC--- t0094891 5

-----------GAGUUAAGUGUCGUUUGUGUCC--- t0605340 1

--ACAAACGACACUUAACACCGC---------- t0661294 1

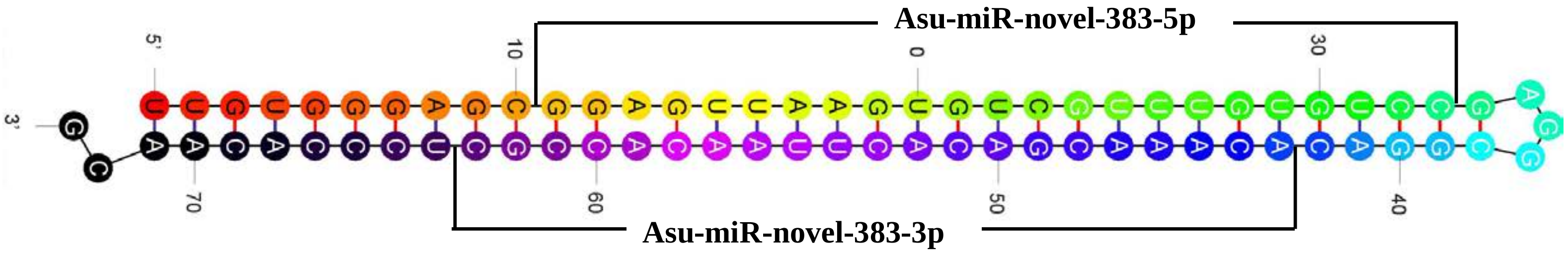

Supplement: Additional file 2: Figure S1 — Detailed blast results of variants and precursors of representative miRNAs in Ascaris lumbricoides and A. suum. [file 1746-6148-10-99-S2.pdf]
